# Piloting the Update: The Use of Therapeutic Relationship for Change – A Free Energy Account

**DOI:** 10.3389/fpsyg.2022.842488

**Published:** 2022-04-11

**Authors:** Gernot Hauke, Christina Lohr

**Affiliations:** Embodiment Resources Academy (ERA) Europa, Munich, Germany

**Keywords:** free energy, active inference, exploration-exploitation-dilemma, therapeutic relationship, cognitive behavioral therapy, safety regulation, emotional survival, embodied cognition

## Abstract

We apply the Free Energy Principle (FEP) to cognitive behavioral therapy (CBT). FEP describes the basic functioning of the brain as a predictive organ and states that any self-organizing system that is in equilibrium with its environment must minimize its free energy. Based on an internal model of the world and the self, predictions—so-called priors—are created, which are matched with the information input. The sum of prediction errors corresponds to the Free Energy, which must be minimized. Internal models can be identified with the cognitive-affective schemas of the individual that has become dysfunctional in patients. The role of CBT in this picture is to help the patient update her/his priors. They have evolved in learning history and no longer provide adaptive predictions. We discuss the process of updating in terms of the exploration-exploitation dilemma. This consists of the extent to which one relies on what one already has, i.e., whether one continues to maintain and “exploit” one’s previous priors (“better safe than sorry”) or whether one does explore new data that lead to an update of priors. Questioning previous priors triggers stress, which is associated with increases in Free Energy in short term. The role of therapeutic relationship is to buffer this increase in Free Energy, thereby increasing the level of perceived safety. The therapeutic relationship is represented in a dual model of affective alliance and goal attainment alliance and is aligned with FEP. Both forms of alliance support exploration and updating of priors. All aspects are illustrated with the help of a clinical case example.

## The Concept of Free Energy

The concept of free energy (FEP) is being used in the context of neuroscience as well as biology, where it has been promoted for its broad applicability and even proposed as a fundamental principle for understanding intelligent behavior and brain function (Friston, 2010). Meanwhile, this principle has also made its way into psychotherapeutic thinking. There are considerable psychoanalytic resonances (Hopkins, 2016; Connolly, 2018; Holmes and Nolte, 2019; Holmes, 2020; Solms, 2021). Smith et al. (2021b) have introduced a computational model of cognitive-behavioral approaches using this principle. For more integrative therapeutic approaches, the concept of free energy has been suggested as a guiding principle for strategic modification of priors (Krupnik, 2019). Overall, interesting ideas are developing under this umbrella, which could provide a common theoretical framework for a wide variety of psychotherapeutic streams (Chekroud, 2015; Duquette, 2017; Papalini et al., 2020; Krupnik, 2021).

Free energy provides a single unified mathematical concept. In all applications, it is concerned with minimizing free energy as a fundamental process of self-organizing systems. Particularly in the face of permanent changes in the environment, this process serves to maintain its sensory states within existing physiological limits. These abstract connections are now to be made plausible with regard to psychotherapeutic questions.

In the course of childhood and depending on experiences of satisfaction or frustration of central needs such as security, belonging, autonomy, self-worth, etc., the brain develops an internal model of the self and the world (Beck et al., 1979; Young et al., 2003; Sulz, 2006). This model contains expectations—they are referred to here as priors—that are so good that the person can survive with them. Development and learning usually lead to a continuous update of these priors. The goal of this process is to continually improve accuracy of the internal model so that the most adaptive behavior possible can be realized even in uncertain situations.

Internal models can never fully prepare for all the uncertainties that might happen. The expectations they generate cannot always represent what then actually happens, so that a greater or less degree of uncertainty must always be expected. This makes discrepancies between what actually happens and what is predicted possible. Such discrepancies are called prediction errors (PEs), and the free energy is essentially the sum of these prediction errors. The free energy principle calls for minimizing such prediction errors and thus minimizing uncertainty. Too much uncertainty, however, contradicts the natural goal of the system to move toward a stable state. Experiences with higher amounts of stress are in many cases necessary to initiate learning processes. Beyond mere tolerance, an additional goal for clients is to learn that they can function even when experiencing stress.

Moreover the intensity of stress must be oriented to the limits of the client—it is unfavorable if he drops out or dissociates. Besides the client’s limitations, duration of such an experience is also a critical parameter. Critically therapy may proceed gradually by working through a hierarchy of situations that are increasingly more urge provoking, to overcome urges and other negative mood states (Craske et al., 2014).

Too much free energy or uncertainty becomes a problem in the long run (Peters et al., 2017). Beyond a certain limit, the physical ability to survive is endangered.

How can free energy or PEs be minimized? Agents can minimize free energy using two strategies. Together, they constitute active inference:

• Update: Agents change their priors, or expectations, to match incoming sensory information. In this way, priors are revised through experience, resulting in more reality-adapted priors.
• Acting: The second strategy is to perform an action to control the sensory input so that the incoming data fit the existing priors best and minimizes PEs (i.e., no updating). In this respect, different exploratory behaviors may be involved.

Active Inference describes perception as a computational compromise between what priors lead us to expect and what we actually experience. That is, perception is shaped by both the internal model and the sensory impressions. This can be described as a form of “controlled hallucination”. What is crucial now is the “mixing ratio” of these two components that finally leads to a perception. If the person is firmly convinced that his or her inner model of a particular situation is correct, that is, that it accurately represents reality, then it has a great influence on the perceptual outcome. In this case, the priors are assigned a strong weight (high precision). At the same time, if low precision is assigned to the current sensory impressions, they have little chance to make a meaningful contribution. In this case, perception is more or less completely under the influence of the internal model; context plays only a minor role.

Whether prediction error leads to an update of priors depends on their relative strength or precision. For example, if someone has repeatedly experienced massive fear toward authority figures, the corresponding prior is “strong.” It dominates experience so much that current information, such as open and friendly behavior of a new boss, hardly matters. This is sometimes referred to as hyper-precise priors, that disregard evidence and alternative explanations that the world is a safe and predictable place (Paulus et al., 2019).

When a patient comes to therapy with his or her problems, his or her internal model has become dysfunctional. It does not respond flexibly enough to changes in the external world, which has been linked to various mental illnesses (Paulus et al., 2019).

When patients repeatedly fail to match what they expect with what is currently happening, a persistent discrepancy, or PEs, occurs when they observe outcomes. They are unable to perform an appropriate weighting of prior expectations and sensory cues. A functional internal model relies on PEs to adjust priors and then appropriate actions. However, in the case of a dysfunctional model, the PEs remain. However, in the case of a dysfunctional model, the PEs remain. Here, this update does not easily succeed, and free energy remains permanently elevated.

## Essential Task of Therapeutic Relationship: Provide New Experience and Support The Update of Dysfunctional Priors

From the point of view of FEP, how should psychotherapeutic relationship be configured so that the client can leave the zone of problematic behavior associated with painful experiences, stress and additional burdensome symptoms? Learning processes must be initiated that allow a healthier adaptation to the environment. Therefore, therapeutic interaction must support the development and exploration of alternative behaviors. In the context of CBT, experimental learning by success is an essential part of many interventions. It is about gaining new insights, reflecting on them and trying them out further, and thereby arriving at a more coherent picture of oneself and the world. At the beginning of therapy, patients are confronted with a lot of uncertainty: “Will I get worse with therapy than before?,” “Can the therapist understand me and how will he react if I fail?,” “Do I have to turn my whole life upside down?,” “How am I going to manage with my job and family?” This experience should be contained and limited in therapeutic relationship. Our client is faced with the decision to what extent he will use the actions and resources he is familiar with for satisfaction of his most urgent needs, which at least guarantees him a minimal degree of security in short term, or whether in the longer term he will have to explore new behavioral paths in order to improve his situation permanently. To get an update of priors, experiments must be designed that realize an appropriate balance between exploring new possibilities and exploiting old certainties. This type of decision-making situation is called an exploration-exploitation dilemma (Wilson et al., 2014). We choose this dilemma here because it links active inference with learning processes in the FEP context. We argue here that the essential task of the therapeutic relationship is to manage the exploration-exploitation-dilemma in such a way that the amount of stress will be limited and so learning processes can happen.

## Common Management of Exploration-Exploitation-Dilemma

The exploration-exploitation dilemma has been addressed in economics, behavioral ecology, and computational neuroscience, where it has a longer tradition. In psychiatry, this approach has only recently emerged (Addicott et al., 2017). How does FEP address this issue?

Consider a person in a particular situation. How will he act? Active inference uses an internal model to develop predicted futures and calculate their free energy. It then selects a sequence of actions (or policy) that will minimize the expected free energy (EFE) (Da Costa et al., 2020). Since we are considering consequences in the future here, we also refer to this as “expected free energy.” If there is no doubt about the expected states, then this reduces to matching the prior preferences with the expected target states. If these target states correspond to rewarding states, then minimizing free energy maximizes expected reward, just as in reinforcement learning.

## Facing Uncertainty: Gathering Information That Is Lacking Lowers Free Energy

In fact, in many situations, the relations are not so clear *a priori*, but ambiguous. In this case, with active inference, the person additionally selects actions that minimize the ambiguity that is present. This results in the fact that he has to collect information. This is done through exploration. At the behavioral level, exploration denotes, for example, search, variation, risk taking, experimentation, play, flexibility, discovery, and innovation. Exploitation takes advantage of all the behaviors that one already knows, feels safe and familiar with, and that provide a proven path to expected reinforcements.

Thus, in uncertain situations, reward-maximizing (or exploitative) behavior is supplemented by novelty-seeking (or exploratory) behavior. This brings us back to the classic exploration-exploitation dilemma. Active inference also solves this decision dilemma by minimizing free energy (Friston et al., 2015). Mathematically, the expected free energy is minimized by combining exploration and exploitation to yield the largest possible amount. Active inference, after all, aims to maximize the evidence for a given internal model and calls for a tradeoff between exploitation and exploration that fits (Kaplan and Friston, 2018). What is clear is that there can be no general or even fixed balance between the two strategies. The two strategies are not independent of each other but are in tension with each other. Those who rely too heavily on exploitative strategies have strong habits, they don’t update their priors. Thus, the person fails to learn more appropriate behavioral strategies that will help him or her cope better with changes in the environment. The other extreme is equally inefficient. The person who engages in excessive exploration makes too little use of past experiences as a basis for later decisions. To be adaptive, however, the agent must continually assess whether to use more or less of one strategy or the other at any given time, depending on the situation (Bruineberg and Rietveld, 2014; Kiverstein et al., 2019a).

In general, uncertain situations are thought to trigger the need for exploration, with the goal of reducing existing uncertainty and promoting rapid learning (Berger-Tal et al., 2014). This finding is likely true in many cases and especially in healthy populations. In fact, however, mental illness appears to be associated with impaired or inefficient exploration behavior (Addicott et al., 2017; Cathomas et al., 2021; Smith et al., 2021a) and several disorders have now been described as involving priors held with excessive precision, including depression and mood (Seth and Friston, 2016; Badcock et al., 2017; Clark et al., 2018), schizophrenia and delusion (Tschacher et al., 2017) and aspects of obsessive compulsive disorder (Kiverstein et al., 2019b). This is exactly what most clients show at the beginning of therapy. In problematic situations, they usually avoid focused exploration, which put them more in touch with their fears. So, therapy will increase FE in the short term. Either they are stuck, clinging to the previous repertoire and avoiding the slightest risks in trying out possible alternatives. Or they lapse into a kind of unsystematic exploration, getting bogged down in this respect and creating too much input and risk. Likewise, dysfunctional oscillation back and forth between these extremes is possible.

**FIGURE 1:**
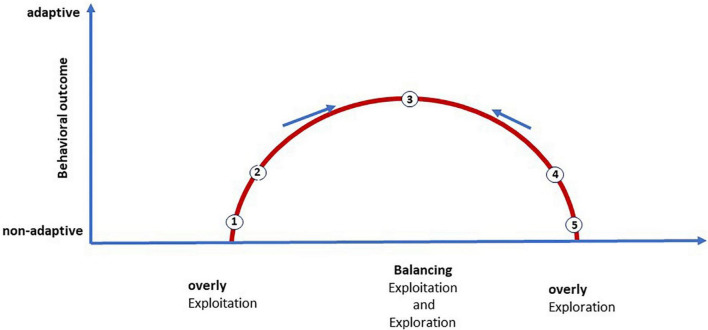
The therapeutic course as an idealized exploration-exploitation-dilemma (red). Goal-directed therapeutic interaction starts at pole (1): Overly exploitation/non-adaptive behavioral outcome: At the beginning of therapy, the client is stuck and repeatedly maintains his dysfunctional pattern; free energy is very high. (2) Elaborating/validating the current model, work with emotions, starting guided reality checks in a safe environment, define experiments. More clarity, first success experiences, less free energy. (3) Balance/more adaptive behavioral outcome: both sufficient safety and sufficient experimentation, ratio may vary, directed exploration, thorough self-directed reality checks. Lowest possible value of free energy (4) Is there evidence that too much exploration, aimless experimenting leads to avoidance of key issues? Problems in therapeutic relationship? Increasing free energy. (5) Overly exploration/non-adaptive behavioral outcome: many new behavioral projects are started but not finished, impulsivity, too much new information, inefficient use of behavioral experiments, no robust learning experience and change, risk of falling back into the grip of the dysfunctional internal model. Free energy is very high again.

A rather vivid illustration of this dilemma for therapeutic purposes emerges when explore and exploit decisions are placed on a continuous scale (Mehlhorn et al., 2015; Addicott et al., 2017). According to this view, decisions can vary along a continuum between overly exploitation and overly exploration. We saw earlier that the most beneficial behaviors occur around a point of equilibrium between the two. These aspects can be found again in Figure 1.

Both “extremes” are problematic in the long run (cf. Figure 1): Obviously, the client remains trapped in a less stable equilibrium if he only engages in exploitation to the exclusion of exploration. He applies his dysfunctional internal model again and again, but learns nothing really new. On the other hand, limiting himself to exploratory strategies may yield too many new undeveloped ideas and too few viable concepts and competencies. He gets bogged down and the results are less certain, so that uncertainty, frustration, and helplessness may again increase.

The therapeutic interaction is thus confronted with the task of determining, always while respecting the client’s limits, an appropriate mix between the two strategies. This balancing act keeps the occurring stress in the change process within limits and minimizes surprise and thus free energy (Figure 1).

## Developing Therapeutical Relationship for Updating Prios

Traditionally, three characteristics of a functional therapeutic relationship have been addressed (Bordin, 1979; Horvath and Greenberg, 1989; Horvath, 2018): (1) development of an affective bond between client and therapist, (2) agreement on the tasks of therapy, and (3) agreement on the goals of therapy. This alliance describes the extent to which the client and therapist are jointly engaged in goal-directed work (Bordin, 1979). These characteristics are summarized in Schulte and Eifert’s (2002) dual working model of the therapeutic relationship. We choose this form of description because it is a good way to incorporate the FEP perspective.

The dual model describes two sides of therapeutic interaction, which can hardly be separated from each other and can often be realized simultaneously (Lundh, 2017).

• Affective alliance: This particular form of interaction must be designed in such a way that the client’s willingness to open up, actively cooperate, try out new behaviors, etc. increases. This establishes an important basis for further change processes in therapy.
• Goal-achievement alliance: This side of therapeutic interaction includes indication and use of specific therapeutic intervention methods for behavior modification as well as joint planning, implementation and evaluation of experiments.

Both aspects of the therapeutic relationship are guided by relationship rules on the one hand and method rules on the other (Table 1):

**TABLE 1:** Examples of relationship rules and method rules.

Rules for affective alliance	Rules for goal achievement alliance
Active listening	Conducting self-directed experiments and expositions
Empathically validating conversation	Tolerating a certain level of arousal and tension
Complementary relationship building	Being aware for bodily signals

In order to described balancing act in the exploration-exploitation dilemma to be successfully accomplished, the client’s internal model of self and world must be worked out at the beginning of therapy. This is the starting point for the first steps toward lowering free energy. A concomitant increase in perceived safety is an essential prerequisite for setting a learning process in motion. In the case described, we encounter a client who is in a state of “over-exploitation” at the beginning of therapy (see Figure 1).

***Case***: *The 28-year-old client, Julia, comes to therapy with depressive symptoms that she has experienced since her teenage years. Julia has been dating Ben for 2 years, and they plan to move in together. Julia works as a dental assistant in a large practice. She is the oldest of four sisters and was used to helping her parents, who are self-employed with a bakery and take care of her younger sisters. She also helps every Saturday with the bookkeeping and orders for the bakery. The parents’ marriage was and still is characterized by quarrels, which scared Julia a lot as a child. Father and mother had little time for the children and demanded, as they did of themselves, daily smooth functioning and a willingness to work hard. “That’s the only way to make it in life,” Julia’s father always said. Julia therefore received a lot of appreciation when she decided a few years ago to study part time beside her job. Since then, she spends every free minute studying. Her relationship with Ben, who prefers to enjoy his hobbies and meet friends in his free time, has deteriorated significantly. He has given up asking her to come along. Julia often feels misunderstood by Ben and withdraws from him more and more.*

**The Trigger for Her Current Crisis**: *Two months ago, Julia’s mother suffered a severe slipped disk and since then can no longer stand behind the counter as much. Julia now works not only on Saturdays in the office but also early in the morning for 3 h at the counter of the bakery before she goes to her own work. She sleeps very little and therefore can hardly concentrate on studying in the evenings. She fluffed two exams and failed. Since then, Julia has had the feeling of losing the ground under her feet. She feels more and more overwhelmed. Physically, she now suffers from severe back pain every day. In the evening, she lies awake ruminating for a long-time despite of being very tired.*

## Stuck in Overexploitation: “Better Safe Than Sorry”

Exploration means choosing an option which has less information, and outcome is associated with a certain risk. Exploitation means staying with an option that one knows well and is believed to produce expected results. Ultimately, it is learning history that determines what choices are made in these situations. At the beginning of therapy, the client relies on what she knows. Following the internal model, we term it as a survival strategy (Sulz, 1994; Hauke, 2018a), refers to the old priors and the attempt to fit incoming data. Her self-regulation is controlled by a survival strategy which, for the purpose of satisfying needs, prescribes relatively narrow behavioral paths by means of its commands and prohibitions. The more stressful the personal developmental conditions were experienced, the smaller the behavioral repertoire. Accordingly, behavioral tendencies used for psycho-emotional “survival” are rigidly maintained to prevent occurrence of the feared. At best, more effort is invested to fulfill specifications of the survival strategy even more precisely. It is like squeezing a lemon that contains no more juice: Quite a bit of effort still yields a few drops. Indeed, it is now well established that both acute and chronic stress leads to the over-selection of exploitative strategies, even though little is accomplished (Lenow et al., 2017). Under the influence of this kind of stress experience, situation at hand is more likely to be appraised as threatening, which furthermore makes selection of exploratory strategies unlikely (Harms, 2017). The maladaptive impact of stress is now what makes stress response no longer context specific.

***Case (Continuation)**: In our case Julia behaves the way she learned it in the strict environment of her childhood. She works harder and does no questioning. Thus, even in current situations, their specificity and differences are not sufficiently addressed. Instead of situation-specific, schematic action is taken. Julia reacts as she has learned in her family. She tries in vain to satisfy her need for appreciation with the same attempts at low-maintenance and performance-oriented behavior. Without need fulfillment, there is a sharp drop in security. The exploration of alternative possibilities of the real adult world is thus omitted, instead the contact to the body is lost more and more: She feels her exhaustion only when she gets a lot of pain.*

This “clinging” to the previous schema is considered by Van den Bergh et al. (2020) in the context of a predictive processing mechanism. The authors develop a perspective according to which many affective and cognitive features (e.g., worry, rumination, impaired anxiety learning, reduced specificity of autobiographical memory, somatic symptoms) of a wide variety of diagnoses can be traced back to a common underlying processing strategy. This they refer to as the “better-safe-than-sorry strategy.” The associated style of information processing is essentially top-down oriented with a tendency toward low sensory-perceptual detail. Here rumination can be seen as a process of maintaining the old dysfunctional priors and policies. Clients also no longer rely on their body signals, for example (Smith et al., 2021c). As a result, bodily signals are suppressed and cannot update the priors by “active inference.” This happens in favor of rapid categorization of threat-related stimuli, but at expense of an update of threat-related priors (Van den Bergh et al., 2020). These priors are held with high precision and as such represent learned critical safety-related aspects. In our specific case, they are all those features that are addressed in the four lines of the survival strategy.

***Case (Continuation)**: The survival strategy is elaborated on the basis of a concrete problem situation (Hauke, 2018a; Hauke and Lohr, 2020), so that it is not only cognitively understandable but also emotionally tangible for the client.*

***Julia’s Survival Strategy**:*

***−Only if I always** perform, function smoothly, and act dutifully*
***−and if I never** enter into conflicts and separate myself from others*
***−then I maintain** security through appreciation and affiliation*,
***−and prevent** losing control and failing.*

Since these precise threat-related expectations dominate processing, noticeable deviations are very often ignored in the current situation. This mode of operation is thus not very context-specific, so that effective error minimization and thus a clear update of priors is omitted. At the same time, active inference generates perceptions and actions that are consistent with prior expectations but do not necessarily match the current situation. Here, this update does not easily succeed, the PEs remain and free energy remains permanently increased. In the longer term, this gradually leads to more and more free energy, i.e., PEs, and ultimately to chronic uncertainty or chronic stress (Figure 1, left). In this respect, a more adaptive internal model can hardly be developed (Van den Bergh et al., 2020).

However, if there are changes in the environment, i.e., if a new context arises, the previously very successful strategies may fail.

***Case (Continuation)**: The context changed dramatically for Julia, when her mother falls ill and she starts working significantly more than usual in the bakery. Due to this overload, Julia experiences a failure of previously successful strategies. This produces massive stress and thus leads to an increase in “free energy”: selected actions no longer produce rewarding energy boost by satisfying the client’s central needs. On the contrary, all threads slip away from her and she is confronted with loss of control and failure—her central fear from line 4.*

This context leads to stronger prioritization of safety, so that a higher-level control is controlled by this prior. It is now provided with stronger precision.

How should client Julia deal with this situation?

## Affective Alliance: The Therapeutic Relationship Provides a Safety Buffer

**FIGURE 2:**
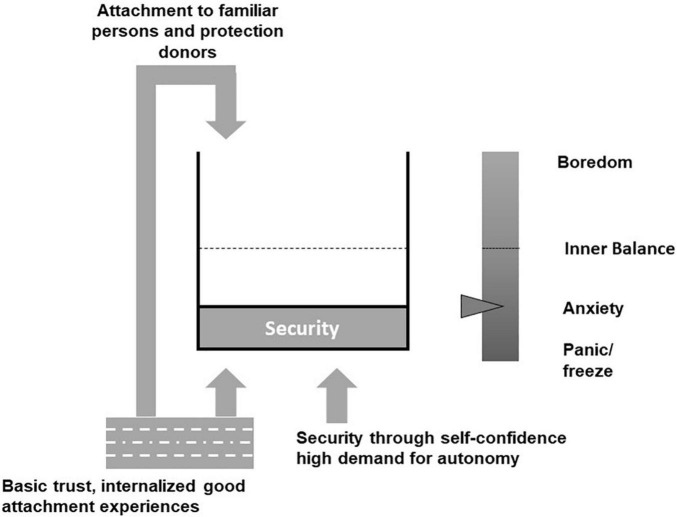
Model of safety regulation modified according to Bischof (2008). The dashed line indicates the optimal level. Here, the person experiences no stress and is in emotional equilibrium.

The client’s internal model is summarized in her survival strategy. It is worked out right at the beginning of therapy and provides a precise “blueprint” for an initial shaping of affective alliance. This is intended to replenish safety reservoir, since the “better-safe-than-sorry strategy” generates stress and thus increasing insecurity and hopelessness due to stagnating error reduction process. To understand these relationships more precisely, let us take a closer look at safety regulation. In respect, it will become clear how satisfaction of various needs is related to the extent of perceived safety. The dynamics of safety regulation are explored in more detail in the Zurich Model of Social Motivation (Bischof, 2008). In Figure 2, the safety reservoir is ideally always well-filled. Its target level is subjective and varies according to personality. If the level is far below the target value, then the person feels too little security and experiences anxiety. Conversely, too much security leads to boredom.

A favorable safety level can be established by means of various sources (Bischof, 2008):

**From the Outside**

Protection and security by close, familiar people, such as partners, dear relatives, friends, appreciative colleagues, etc.

**From the Inside**
(1) By internalized good experiences of attachment. Because of positive early childhood experiences, “basic trust” is formed. This corresponds to an internal working model of secure attachment.
(2) Security based on self-confidence, which in turn is associated with high autonomy needs.

We are dealing here with two major sets of needs (Epstein, 1994; Mikulincer and Shaver, 2004; Grawe, 2007):

• Needs related to attachment and social relationships, e.g., security, belonging, caring, harmony, feeling valued and welcome, etc., as well as
• Needs related to the self, e.g., self-worth, self-actualization, expansion of the self through the exercise of power, through new experiences, knowledge, achievement.

Looking at the client’s survival strategy, we can see in the third line, strong attachment-related needs. Her willingness to perform should make her attractive as an attachment partner, i.e., she fills her security reservoir primarily by satisfying needs that can be thematically assigned to the attachment motivation.

Based on our client’s needs profile and Figure 2, it is clear that an increase in the level of felt safety is achieved by the therapist satisfying the specific attachment-oriented needs for being seen and belonging in the therapeutic relationship.

***Case (Continuation)**: Julia’s therapist gives her a warm welcome from the beginning and asks her what she needs to feel comfortable in therapy. She expresses appreciation for her willingness to take on responsibility, shows empathy for her exhaustion and tireless dedication to the family business. In addition, the therapist acknowledges Julia’s commitment to therapy. For here, Julia’s dutiful nature is also very helpful, as she also practices therapy-promoting behavior regularly and therefore achieves success quickly, e.g., improvement of her sleep quality.*

This puts the client in a realm of experience—a kind of ecological niche—in which her internal model receives sensory input that it in turn predicts as probable. This leads to a lowering of uncertainty or free energy.

The successful shaping of affective alliance depends both on accuracy with which the therapist grasps her client’s need structure and, on her caution, and flexibility in acting on it in a need-satisfying way with her behavior. Here, the application of relationship rules is particularly important (Table 1). By designing the relationship complementary to her needs, the therapist provides experience that correspond to the expectation or priors of the client. In this respect, the therapist tries to actively establish and reinforce goals and experiences that are outlined in the survival strategy. Conditions and experiences that the client perceives as bad or avoids are not activated for the moment, if possible.

***Case (Continuation)**: In the first phase of therapy, Julia’s therapist therefore avoids questioning her client or criticizing her approach. This was a fear that Julia had also expressed at the very beginning of therapy. She said at the time, “I’m afraid that you (the therapist) will just say that I shouldn’t help my parents anymore and finish my studies. But it’s not all that simple for me.” Julia experienced it as very beneficial to be understood and seen with her needs in this way. This allows her to open up more and express the full extent of her stresses.*

In principle, complementary relationship design shows empirically high effectiveness in terms of therapy outcome (Caspar et al., 2005). With this form of relationship design, the therapist creates space for processes of active inference according to client’s previous familiar pattern. This enables him or her to adopt a PE-minimizing and thus self-informing policy. In other words, this strategy for minimizing free energy looks like this: The therapist engages with the client in a dance in which the client leads and the therapist responds in ways that the client expects.

## Establishing the Goal Achievement Alliance: Leaving the “Problem Zone” With Directed Exploration

The goal-achievement alliance serves to reduce the problem behavior itself by means of effective methods as well as to promote goal behavior (Schulte and Eifert, 2002). In our chosen perspective, this means establishing and supporting directed exploration. The goal achievement alliance can be labeled in therapy as a kind of expedition, in the course of which difficult situations have to be mastered but also important discoveries can be made. This requires a shared map, good preparation, and a coordinated approach.

Typically, people use a mix of undirected and goal-directed exploration (Wilson et al., 2014; Schwartenbeck et al., 2019). Goal-directed, information-seeking exploration is guided and motivated by uncertainty in the agent’s world model. This means that agents typically selectively choose options that are informative, i.e., those associated with greatest uncertainty. Undirected exploration is defined as a deviation from current most valuable strategy by unplanned selecting a different option. What does this mean for therapy? Clients are unlikely to spontaneously choose the option associated with most uncertainty. Nevertheless, targeted experiences must be provided to selectively help erase uncertainty in this respect. To address client’s fear/uncertainty, experiments are constructed along a difficulty hierarchy. The client is encouraged to “question reality” and is supported in trusting his or her own experience. Undirected exploration—understood as a client’s unplanned and impulsive actions—should be limited at the outset so that what has been achieved is not risked carelessly. Once a certain level of safety has been reached, then undirected exploration can later lead to a pleasurable journey of discovery.

## Directed Exploration Aims at the Exploration of the Self and the Behavioral Niche

The previous outline of directed exploration will now be made more concrete. Directed exploration means searching for and reflecting on specific information. This information concerns both client’s view of himself and his view of the world: Who am I and how do I react in a certain situation, what are my emotions and impulses for action? On the other hand, clients need information about the efficacy of their actions, possible reactions of their environment, and an assessment of how they are likely to cope with consequences of their actions. Typically, the internal model includes beliefs about future states and policies, with the most likely policies leading to preferred outcomes. In this sense, behavior is also understood here as fulfillment of optimistic predictions (Friston et al., 2016). Clients who are asked to test new situations do not necessarily share this optimism, as they lack adaptive priors about future states, effective policies, and clarity about preferred outcomes. Here, importance of therapeutic interaction in establishing optimism and hope becomes clear. The updating process should be handled in a manner which is free of judgment and within the context of a benevolent error culture that promotes learning. In the service of an acceptable level of certainty and controllability, the goal-achievement alliance provides the following methodological rules: (1) selection of an appropriate small-step approach, (2) joint determination of the level of stress to be tolerated, and (3) development of plans and strategies to prepare for emerging uncertainties. The precise content of these aspects is again guided by the model of safety regulation specific to the client (Figure 2).

In order to keep the free energy during exploration reasonably limited from the very start, goal-achievement alliance additionally provides for a limited selection of future-related priors. These are obtained based on a variety of tasks, joint observations, and hypotheses. To this end, we thematize two foci of work for the goal attainment alliance: (1) emotional work and (2) behavioral experiments.

## Exploring Emotional Events: Emotional Work Refines the Internal Model

Emotional experience can be understood as a somatic change that occurs in response to stimuli that affect our physical or mental well-being (Pollatos and Ferentzi, 2018). More current theories of emotion focus primarily on the perception and recognition of various bodily changes, although traditional basic assumptions such as “appraisal” may also be included as important components of emotion. Within their integrative model, Smith et al. (2018) describe three processes:

(1) Current situations, thoughts, and memories elicit changes in the brain and body (e.g., changes in attention, changes in heart rate, etc.).
(2) Physical changes are registered and classified in terms of causation (e.g., I feel shaky because I drank too much coffee or because I am scared).
(3) Finally, it is decided to what extent attention is paid to such feelings.

Appropriate evaluations are based on learning experiences. Even if bodily feelings are present, they may remain implicit because, for example, it was “forbidden” in the learning history to feel and express feelings of being overwhelmed. The model highlights the importance of interoceptive processes for emotional experience. Namely, interoception describes the perception and representation of internal body signals (e.g., heartbeat, breathing activity, feelings of satiety). It contributes to body awareness, defined as attentional focus on and awareness of internal body sensations, and is critically involved in the regulation of emotions (Pollatos and Herbert, 2018).

How is this conceptualized within the framework of FEP?

In order to represent emotional events, the internal model includes interoceptive priors in addition to exteroceptive priors. They result from previous experiences of similar situations. They form the basis not only of predictions about upcoming interoceptive and exteroceptive signals but also about the most appropriate action to deal with the upcoming sensory flow. All of this is processed in a single inference process (Seth, 2013). In this respect, these predictions are compared with sensory input from body and context. Minimizing the prediction errors results in the perception of body sensations and action impulses. The meaning emerges by mapping the response patterns to learned emotion concepts (“this feels like anger”). However, minimizing PEs or free energy in this respect may encounter problems (Smith et al., 2020):

(a) Emotion concepts are too diffuse and provide inaccurate predictions regarding body feelings, context, and action,
(b) There are hyper-precise priors that dominate interoceptive and/or exteroceptive perception,
(c) Learning history has caused selective attentional biases that now ignore bodily or contextual cues.

All these questions are addressed by our work in the so-called “Emotional Field” (Hauke and Dall’Occhio, 2013; Hauke, 2018b). In this respect, two important methodological rules of the goal achievement alliance are applied. They refer to the practice of focusing attention on the body and maintaining an agreed-upon level of negative affect. We repeatedly encounter clients who have difficulty naming emotional categories. This is facilitated if attention is first selectively drawn to body signals when an emotional episode occurs. Subsequently, this can be reflected upon and usually emotion concepts can be named and reflected upon (see Duquette and Ainley, 2019). Our working format is essentially bottom-up and provides for a clear separation between experiencing specific bodily sensations and talking about them. Holmes and Nolte (2019) arguably refer to the same thing when they speak methodologically of “decoupling.”

***Case (Continuation)**: In the Emotional Field Julia works with her therapist on a problematic situation with her father. The argument arose when Julia wanted to explain to him that she cannot help out in the bakery on Saturdays as well because she needs time to study. The father, however, had shown no understanding at all and insisted that he needed her for bookkeeping on Saturdays as well. In order to ensure an intensive emotional activation of Julia, she is first asked to portray her father as vividly as possible. For this, Julia, guided by her therapist, chooses a typical body posture, a frequently heard phrase and the corresponding facial expressions. So, Julia’s father becomes tangible as the central reference person in the room. Julia then positions herself in front of the wall that shows her “visualized” father. Julia chooses a middle distance and a smaller position, because she has always felt inferior to her dominant father, although he is physically much smaller than she is. Supported by the therapist, Julia now begins to describe how she feels in her body. Julia reports a heaviness in her chest that makes her breathing shallow. In addition, she feels an impulse to make herself even smaller. Julia should now give in to this impulse. She crouches down very tightly and can hardly breathe. Julia should now wait for the next body impulse and give in to it. After a short time, she reports a heat rising in her head and a tension in her arms. Julia feels the urgent desire to get up. When Julia stands in front of her father again, however, heat and tension immediately disappear. Julia wants to make herself small again. At this point, the therapist asks Julia to move to the so-called expert position (Hauke, 2018b; Hauke and Lohr, 2020), a reflection point in the room located away from the experience space in front of the wall. Here Julia and her therapist try to give a name to the different emotional states just experienced. The first emotion that emerges is very familiar to Julia. It is fear, which takes away her breath and helps her to hide. Julia is familiar with this strange heat, but she cannot think of a more concrete name for it. The therapist writes down the two terms fear and heat on one note each. Julia places both pieces of paper accordingly in front of her visualized father. After this short conversation, the therapist asks Julia if she feels ready to go into the experience again and find out more about this heat. Julia agrees. Both start again with the confrontation of Julia in front of the wall with her father. As in the first run, Julia’s fear shows up first, which—if intensified—again creates erection impulse, heat in the head and tension in the arms. The therapist asks Julia to reinforce these body signals as well. Julia now also feels a strong desire to move closer to her father and reports an impulse to yell at him. She yells, “You bully make us all sick!” Now Julia feels significantly taller. To reinforce this feeling, her therapist gives her a wooden stool to stand on. From here she can now look down on her father, but at that moment all courage leaves Julia and she immediately wants to make herself small again and back away. The therapist lets Julia follow her body impulses and then discusses this with her again on the expert position. In this way, Julia experiences her survival strategy first hand. The body impulses take up a lot of space and make it easier for Julia to understand what happens to her in conflicts. The so-called reaction chain (Hauke, 2018b) becomes apparent (Table 2).*

**TABLE 2:** Julia’s reaction chain.

Reaction chain	Julia’s reaction
Triggering situation	Father has no understanding and demands her work performance
Primary emotion	Anger
Primary impulse	To attack—to yell at the other person
Anticipation of negative consequences	losing control, losing the other person’s favor
Secondary emotions	fear
Secondary impulse	Julia wants to make herself small
Visible behavior	Julia gives in and does the work anyway
Symptoms and problematic consequences	Feelings of inferiority and exhaustion

*Again, and again, Julia goes back to her experience, and thus shame and guilt gradually become apparent as further emotions associated with this situation. By working in the Emotional Field, Julia understands better why it is so difficult for her to stand up for her needs in everyday life. Because not only her fear but also shame and guilt slow her down in anger. She has a strong trio of stopper feelings (secondary emotions), which ensure that she behaves in accordance with her survival strategy.*

*In the first contact with her anger Julia was not even able to recognize or name it. She also felt the power that was connected with the anger and this awaked her interest. The special thing about this kind of guided confrontation is that Julia is first enabled to learn about the emotional basis for her new behaviors and this bottom-up. So, she learns not only from her mind that it would be important to assert herself, but she feels her anger and so the associated power enables her to assert herself. It is her primary emotion that becomes palpable to her as a new sensory input from the combination of her needs and conditions prevailing in environment—namely, her unyielding father. Experiencing the secondary emotion of fear makes it clear to her how strongly her existing internal model has a grip on her. Consistently directing attention to bodily sensations is the key here, as it spatially separating reflection from experience during therapeutic work. Julia understands and feels how her survival strategy prevents problem solving in present. She can experience at the same time that everything she needs emotionally for a solution is already present within her. The central experience is the tipping into each other of primary and secondary emotions. These are the best prerequisites for successfully mastering later behavioral experiments. Julia will not only be able to rationally comprehend them as an external suggestion of an expert, but also experience them as an authentic impulse from herself. And this despite the fact that it is diametrically opposed to her existing model of survival strategy.*

The method rules listed in Table 3 help to adapt therapeutic behavior to the two different settings, in particular to strengthen the relationship and trust in body sensations as early warning signals (Pollatos and Herbert, 2018; Weineck, 2018).

**TABLE 3:** Method rules for working in bottom-up and top-down settings (Hauke and Lohr, 2019).

**Bottom-up oriented work**. - Focuses on sensory perceptions, body sensations and impulses, movements of the whole body and parts of the body in space, - Observes to access roots of emotional experience, automatic impulses and pre-linguistic processes, - Induces sensorimotor input, e.g., by touching, handling, tensing, moving, conscious breathing, etc., to make automatic responding conscious. - Time perspective: focuses on moments of the here and now, creating opportunities to sense impulsive behavioral responses as they occur, to resist automatisms, and to try out alternatives. **Top-down oriented work**. - Seeks linguistic expression for an experience, provides interpretation and often alternative interpretations of experiences, - Records and verifies beliefs, compares, relativizes and communicates the experience. - Devises solutions to problems, goals, as well as plans and intermediate steps, their timing, etc., formulates a clear intention. - Time perspective: covers time span past to future. Placing experiences in life history, determining their significance for present and future, identity formation.

## Directed Exploration by Behavioral Experiments

The FEP perspective refers to testing new situations as “active learning,” where uncertainties in the internal model are reduced by observations and true attributions (Schwartenbeck et al., 2019).

This is done using directed exploration guided by the contents of the survival strategy. This selection of targets restricts the client to behaviorally relevant trajectories, reducing for the moment the variety of data a model must consider. Using FEP, it can be shown that efficient action-oriented models can be learned by now combining goal-oriented and exploratory (information-seeking) behavior (Tschantz et al., 2020).

Concrete action is designed to provide corrective experiences. “Providing corrective emotional experiences” is considered one of the recognized common factors of psychotherapy (Tschacher et al., 2015).

The focus in this respect is on developing and testing behavioral alternatives that may be more adaptive in terms of need satisfaction. In this respect, regarding the balance aspect in Figure 1, the following applies: preserve the good and dare the new. Exploitation and exploration are thus placed in a dialectical relationship to each other. This dialectical attitude is also found in modern CBT, where it is referred to as the acceptance principle (Fruzzetti and Fruzzetti, 2008). As described above, the minimization of free energy depends on the ability to control directed exploration and exploitation in such a way that the perceived stress can still be well tolerated. In constructing and executing alternative approaches, the client can take familiar and proven elements of her previous exploitative strategies and combine them with the novelty of her directed exploration. The mutually determined mixture ratio is oriented to the fact that the client can still well bear the possibly occurring fear. Thus, the goal-achievement alliance conveys an essential moment of control. Directed exploration contains, depending on the intention, a certain degree of uncertainty that cannot be avoided. Experimentation can involve more or less risky events that create a massive prediction error and thus a lot of free energy. The goal attainment alliance can provide a key skill here, which we will refer to in this context as “competence in dealing with uncertainty.” This requires that failures be realistically calculated in. This is not looking through rose-colored glasses, because here the expectation of success is contrasted with possible problems in achieving the goal. That such a view—one could call it functional optimism—is more conducive to goal realization has been empirically proven by Oettingen (1996). According to this, the frequently praised positive thinking is not very promising. It only contributes efficiently to goal realization if it is simultaneously compared with a possible negative reality. This more problem-oriented view has an attention-diverting effect and, in contrast to blue-eyed positive views, provides clues as to what needs to be paid closer attention to and what may also need to be avoided; the feasible is separated from the unfeasible (Oettingen and Gollwitzer, 2010). In a further step, therefore, the behavior plan is still examined for possible failures using a strategy of mental contrasting and then supplemented. The practice of such competencies leads to more and more priors about appropriate policies, so that the client will minimize the free energy in the future by an appropriate policy selection process.

The client may drop out of the goal-achievement alliance, become involved only in a very non-committal way, and instead of directed exploration, just experiment aimlessly (Figure 1, 4). She may have reached a point where she is confronted with a difficult issue that she wants to avoid and distracts herself with activism, but also wants to please the therapist somehow. Stress and free energy rise again, and she may find herself trapped in old patterns again (Figure 1, 5).

**FIGURE 3:**
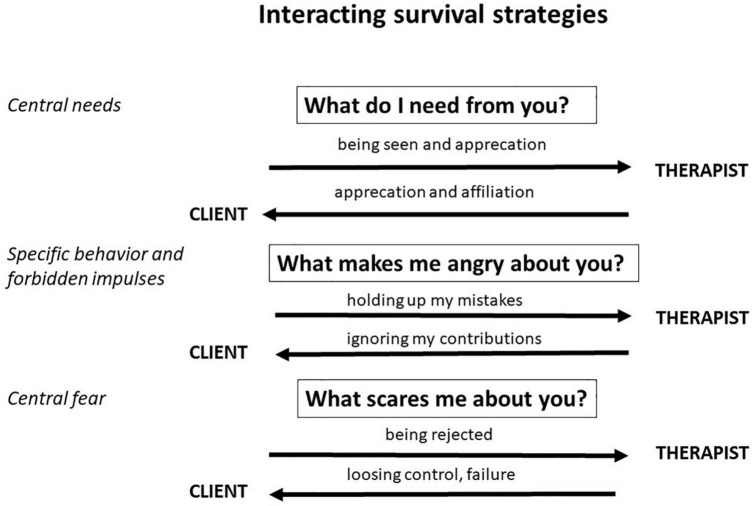
Interacting survival strategies between Julia and her therapist.

*Case (Continuation): Directed exploration in practice means that client and therapist work together on a goal and the plan to achieve it. This involves a successful mixture of old and successful safety regulation from the known traces of the survival strategy as well as a targeted testing of new behaviors. After working on survival strategy and reaction chain, Julia knew her own old patterns very well. As she worked on her anger and the strength that came with it, Julia learned more about the motor for her new behaviors. One evening, Julia left the therapy session full of energy and drive after a successful anger confrontation. She had apparently ignored the therapist’s recommendation to think more carefully in the coming week about how she could use this anger to help solve her issues without giving up everything that is important to her. The therapist’s goal was to keep the client on the path of directed exploration so that she could concretely choose the goal with her in the upcoming session and plan a suitable strategy for achieving it. However, following her anger impulse, Julia drove to the bakery on the same evening, where her father was busy preparing for the next day. With this impulsive action, she left the path of directed exploration (Figure 1, 3) and landed at the point of undirected exploration (Figure 1, 5). Without thinking about the consequences, Julia unloaded all her anger and yelled at her father. She accused him of wrecking her with his bossy manner. But her father did not put up with Julia’s behavior. He also yelled at her and accused her of being ungrateful. It was only because of his hard work that she could study at all and have the life she was currently leading. As usual, he appealed to her guilty conscience. It worked well again. Julia could not counter her father’s accusations and was suddenly struck by feelings of fear, guilt and shame. She fell right back into the other extreme of overly exploitation (Figure 1, 1). She apologized and backed down.*

*In the next therapy session, she reported the incident to her therapist, who reminded her again of her recommendation at the end of the last session. This time, Julia acquiesced and set her goal: She would like to help out at the bakery only 2× a week instead of 5× in order to have more time for her studies and her relationship. The therapist also worked out a conversation guide with Julia on how she could talk to her father in peace and also prepared her for possible difficulties in the conversation with role-playing. She wanted to talk to him after lunch on Sunday and planned 30 min for it. In this way, a good mixture between well-tried paths and new behaviors emerged, which enables Julia to achieve her goals without generating too much free energy and thus stress.*

## Frustrating Duet: Working Directly With the Therapeutic Relationship

In social situations, successful communication depends on people aligning their mental states with those of their interaction partners. We speak of cooperative communication when the participants succeed in aligning their mental states about events in their shared environment. This concept of alignment is found in developmental psychology and behavioral biology (Tomasello, 2008). The therapeutic relationship is just that: developing and establishing a cooperative relationship through affective and goal attainment alliance. Active Inference assumes that a person uses a predictive model of what they would expect from a social interaction in terms of emotional content, what alternative emotional content would predict in the auditory or visual domain, and what a change in those expectations would do (Demekas et al., 2020). This is echoed by Vasil et al. (2020) and presented in the Free Energy framework. According to this, humans are endowed with an adaptive prior that controls this alignment. Accordingly, the selection of action strategies depends on providing sensory evidence that one’s mental states match or are at least similar to those of the interaction partner. This adaptive prior guides actions and inferences. Specifically, the directing of attention occurs in such a way as to gather evidence for this mental match. Cooperative communication in this respect emerges in a process of convergence to shared internal models (Friston and Frith, 2015). This ideal typical progression of the process of agreement occurs in healthy interaction partners in the best-case scenario and may not be fully realized in the client-therapist relationship until the end of psychotherapy. Clients who enter the psychotherapeutic relationship with a better-safe-than-sorry strategy are certainly concerned with alignment. They strive to produce sensory evidence that conforms to their model, priors, the imperatives and prohibitions of their survival strategy, respectively. That is, their interactive world is *a priori* confined to a limited sensory field. However, this usually has little to do with the real person of the therapist and is, after all, primarily oriented to the prior for safety. If the therapist develops empathy for the cognitive-affective state of this safety-oriented strategy, she is able to provide signals that fit into this narrow sensory field. This has the effect of minimizing the amount of PE and lowering the free energy. This is consistent with the complementary relationship design discussed earlier. As therapy progresses, however, the goal is to expand this sensory field with the help of new experiences and the development of alternative priors. A particularly efficient way is represented by the work with the therapeutic relationship. However, since this touches the previous “secure basis” of the relationship formation, this developmental step is only indicated in the last third of the therapy. The previous secure basis was created by the therapist largely serving the client’s priors. Here an emancipation of the client can be achieved by making the real therapeutic relationship visible and exploring it together. This begins with a frustration.

*Case (Continuation): In the last part of therapy, it is often useful to include the therapeutic relationship in the learning process. The goal is to achieve a transfer of what has been learned so far to this particular relationship as well. Julia was making good progress in therapy and so it seemed appropriate to also explore the current state of the therapeutic relationship together. So far, Julia was used to her therapist appreciating her dutiful nature, encouraging her, and meeting her needs for appreciation and attachment. From now on, the therapist took back more and more her complementary relationship design, in which she took the lead in this “duet.” She presented herself in the next therapy session as a factual rather questioning counterpart and reacted less understandingly than usual. The client had just told that she had helped her sister move. Julia was visibly irritated and seemed unsettled by the therapist’s questions, whether it was really okay for Julia to let her sister help her move while she was still suffering from back pain and had been helping out so much at the bakery. Julia didn’t quite know what hit her. She felt an unusual lack of understanding in her relationship and that caused stress. She didn’t know how to deal with it, seemed irritated and kept silent. The therapist suggested to use this as an opportunity to take a closer look at the therapeutic relationship and to present it with Julia in the room.*

## Seeing Is Believing: Embodiment of the Relationship

For this purpose, the therapeutic alliance is represented in space. By positioning the bodies in relation to each other, the different aspects of the relationship become visible and can be felt very quickly. Using a technique that utilizes insights into the embodiment of power and psychological closeness (Schubert et al., 2009; Davis et al., 2011), the client is encouraged to represent the therapeutic relationship in space. Such a representation quickly gets to the heart of the quality of the relationship while not being too confrontational (Hauke et al., 2016). Abstract concepts of power and psychological closeness/distance are made visible in space with the help of horizontal and vertical distances. The corresponding sensory input is of high precision and may be in competition with the priors of the internal model. In this respect, the previous dysfunctional interaction style, which is also evident outside of therapy, usually becomes very apparent. At this point, the relational work can leave behind the exploitative style and become somewhat more exploratory. Exploration is supported here by the therapist resisting the implicit invitation that arises from the patient’s habitual priors. The resulting interaction with the therapist is therefore contrary to the client’s expectations and creates uncertainty. Uncertainty increases because the therapist has consciously created—at first gradually—a situation that resists the previous dysfunctional mechanisms of alignment.

*Case (Continuation): Julia began, guided by the therapist, to present the relationship from her point of view. Julia feels inferior to her therapist—she therefore makes herself much smaller and squats down while asking the therapist to stand on the stool. She also feels dependent on her goodwill and is very afraid of doing something that could endanger it. She therefore kneels directly in front of her and looks at her from below, so that she is able to follow her reactions in gestures and facial expressions. Asked about how she feels, Julia says, that her position on her knees feels uncomfortable but also familar.*

*The therapist asks Julia to try something new out. Julia agrees. The therapist gets off the stool and increases the distance between Julia and her. She takes a rope which she had deposited within reach and offers Julia to grab the other end. Julia grabs it. The therapist shortens the rope so that Julia has to stand up to stay in contact with the therapist. Both are now standing in front of each other with a distance of about 1.5 m. This was how the therapist experienced the first half of their therapy together. Her primary goal was to strengthen Julia and, in the meantime, she sees her at eye level with all her new abilities. But then the following happened: The therapist reaches into the neighboring bookshelf and hands the client one book after another. The client stumbles while trying to keep five books in under her arm. The therapist asks Julia how she feels: Julia states that it felt unusual but good for her to get on eye level with the therapist with the help of the rope. But now she feels overwhelmed and frustrated. The books are hard to hold, she says, and her shoulders are already aching. She has known this feeling for so long. All the burdens and obligations are just too heavy, she says. The therapist explains to Julia that this is exactly what she wanted to achieve with her critical questioning, namely that Julia directs her attention to the burden of her duties—here represented by the burden of too many books. Julia begins to understand how important critical inquiries are for her. Since she falls into old patterns again. Julia and her therapist start talking about what alternative actions she could try out right now. Julia puts down the rope to be able to carry more books. But without the connection, she feels uncomfortable and the load is still too heavy for her. The therapist asks Julia what else she could do to lighten her load without taking off the rope. Julia hesitantly begins to understand: There are just too many heavy books. Julia feels a distinct increase in tension at the thought of putting down books she has accepted. The therapist encourages her to try it out. One book after the other moves to the floor. A first positive experience for Julia in breaking her survival strategy in the therapeutic relationship. She successfully shed burden when pointed out without having to give up connection to a significant other. On the contrary, Julia’s therapist gives her a lot of credit for choosing to put down the book load. Julia feels a definite relief, and the pain in her shoulders slowly disappears. The foundation for the next level in therapeutic work is prepared, in which Julia learns to deal with frustrations within the therapeutic relationship as well.*

## Therapeutic Relationship in Supervision

During therapy, the client’s survival strategy starts interacting with the therapist’s one. This can unintentionally lead to a permanently frustrating duet. Therefore, regular supervision is an important factor for high quality in therapy. Using the already familiar case example, we would like to briefly outline this point and also embed it in the concept of free energy.

Security in therapeutic relationship is of utmost importance in order to update the client’s priors, reduce her uncertainty (Figure 1, 1 and 5) and to help her more and more to achieve a balance (Figure 1, 3). However, this is only possible if the therapist is also able to her own priors during the therapy that she can be like a rock for the client. Usually it depends on the survival strategy of the therapist in which way this can become a challenge at different points of the therapeutic process. This challenge arose for the Julia’s therapist toward the end of therapy:

*Case (Continuation): Julia’s therapist herself comes from a family with self-employed parents. Her father works as a country doctor and her mother supports him as a physician’s assistant. The family climate was also characterized by a lot of pressure to perform and hardship. The father hoped that her twin brother would 1 day take over the practice and always demanded top grades from him in school. Her brother, on the other hand, was very musical and preferred to become a pianist. This led to strong conflicts in the family, in which she often took on the role of a mediator and tried to appease her father.*

*Survival Strategy of Julia’s Therapist:*

*−Only if I always do my best, anticipate the needs of others, and act diplomatically*
*−and if I never make mistakes or show weakness*
*−then I maintain security by being seen and appreciation*,
*−and avoid being rejected.*

*The therapist therefore found it very easy to meet Julia’s needs in terms of complementary relationship building and thus quickly establish a safe climate that reduced “free energy.” However, it became much more difficult in the last third of treatment, in which it was a matter of pointing out Julia’s maladaptive relationship management a bit more clearly.*

*Here the therapist sensed a real inner blockade to frustrate Julia in her needs. Because of her own life story, she could empathize all too well with Julia’s stress and suffering. So, she went to supervision with the following question: How do I manage to get into a good directed exploration with my client despite my own patterns?*

*In supervision, the first step is to visualize the interaction of the two survival strategies. The emerging mutual expectations and fears, could be represented as follows:*

*In this (Figure 3) it becomes visible at a glance at which point it becomes difficult for Julia’s therapist to keep her own free energy low: Namely, when she realizes that the therapeutic process now expects her to actively frustrate her client. Her own survival strategy immediately anticipates the occurrence of the central anxiety. She fears Julia might drop out of therapy or not open up to her anymore. If the therapist remains faithful to her survival strategy due to her own high stress (Figure 1, 1), she would simply continue to meet the client’s needs in a kind of anticipatory obedience. This is exactly where supervision intervenes, because this approach would prevent the client’s developmental process in the sense of directed exploration and jeopardize therapeutic progress. Through the trusting relationship with the supervisor, the therapist can update her priors and work with her not to avoid the client’s frustration but to actively enter into the clarification of the therapeutic relationship and thus begin the directed exploration. To do this, the therapist risks confronting her own anxiety and learns to endure it in favor of her therapeutic role. In this way, the circle of successful therapeutic work is closed by supervision, which also enables the therapist to repeatedly establish a balance between exploration and exploitation with his or her own patterns in daily work.*

## Conclusion

To our knowledge, this is the first time that the CBT process piloted by therapists has been seen through the lens of the free energy principle (FEP) and vice versa. In particular, viewing the therapeutic process in the format of the exploration-exploitation dilemma offers in this respect both novel and important perspectives with regard to the successful management of this process.

Patients’ problems can be seen as priors that do not update and which keep supressing the PEs that are produced by new incoming data. In view of FEP, CBT can be seen as a method that supports an update of the internal model by revising the priors, thereby minimizing free energy. From this perspective, CBT interventions that promote this will be helpful. We have shown that in this respect, the promotion of agency in particular, as well as the design of opportunities for extended sensory and interoceptive sampling in the context of directed exploration, are of central importance.

The quality of directed exploration and the updating of priors depend critically on the developmental space opened by the therapeutic relationship, conceptualized here as an affective and goal-achievement alliance. FEP provides clear measures for assessing these alliances and their effectiveness.

Crucially, in this respect, therapists must also keep an eye on their own stress in this dyad, e.g., in the context of supervision. Otherwise, interaction problems may occur.

The more free energy there is, the greater the uncertainty experienced, which simultaneously results in an increase in metabolic energy costs for the brain (Peters et al., 2017). Here, it could be useful to adapt energy-demanding therapeutic interventions more precisely to the patient’s current life situation and thus orchestrate the course of therapy more successfully.

## Data Availability Statement

The original contributions presented in the study are included in the article/supplementary material, further inquiries can be directed to the corresponding author/s.

## Author Contributions

Both authors listed have made a substantial, direct, and intellectual contribution to the work, and approved it for publication.

## Conflict of Interest

The authors declare that the research was conducted in the absence of any commercial or financial relationships that could be construed as a potential conflict of interest.

## Publisher’s Note

All claims expressed in this article are solely those of the authors and do not necessarily represent those of their affiliated organizations, or those of the publisher, the editors and the reviewers. Any product that may be evaluated in this article, or claim that may be made by its manufacturer, is not guaranteed or endorsed by the publisher.
